# The natural history of body-first versus brain-first Parkinson’s disease subtypes

**DOI:** 10.1007/s00415-025-13050-y

**Published:** 2025-04-09

**Authors:** Vittorio Velucci, Angelo Fabio Gigante, Giovanni Iliceto, Roberta Pellicciari, Barbara Vitucci, Sarah Idrissi, Marcello Mario Mascia, Antonella Muroni, Tommaso Ercoli, Paolo Solla, Giovanni Defazio

**Affiliations:** 1 Department of Translational Biomedicine and Neuroscience, University of Bari Aldo Moro, Piazza Giulio Cesare 11, 70124 Bari, Italy; 2 Section of Neurology, San Paolo Hospital, Bari, Italy; 3 Neurophysiopathology Unit, Polyclinic General Hospital, University of Bari Aldo Moro, Bari, Italy; 4 Neurology Unit, University Hospital of Cagliari, Cagliari, Italy; 5 Department of Neurology, University of Sassari, Sassari, Italy; 6 IRCCS Neuromed Institute, Pozzilli, Italy

**Keywords:** Parkinson’s disease subtypes, Body-first, Brain-first, REM sleep behavior disorder (RBD), Premotor symptoms, Motor progression

## Abstract

**Background:**

Several lines of evidence support the hypothesis of brain-first and body-first Parkinson’s disease (PD) subtypes, characterized by distinct origins of α-synuclein pathology. However, data on premotor non-motor burden and motor progression in these subtypes remain inconsistent.

**Objective:**

To analyze the natural history of body-first versus brain-first PD subtypes.

**Methods:**

Data from 400 PD patients enrolled at a single Italian center were analyzed. All patients underwent a standardized retrospective baseline assessment of premotor and motor symptoms at onset and were prospectively followed. Premotor REM sleep behavior disorder (RBD), considered a prodromal phenotype of the body-first subtype, was used to divide patients into two groups: 81 patients with probable premotor RBD (PD^preRBD+^) and 319 patients without (PD^preRBD−^).

**Results:**

At motor onset, PD^preRBD+^ patients were older than PD^preRBD−^ patients, exhibited less tremor, and more frequently presented with bilateral motor symptoms. PD^preRBD+^ patients also reported a greater burden of premotor symptoms, including hyposmia, cognitive impairment, pain, constipation, and other dysautonomic symptoms. Over the follow-up period, PD^preRBD+^ patients progressed more rapidly to Hoehn and Yahr stage 3, even after adjusting for sex, years of schooling, age at motor onset, and initial motor phenotype.

**Conclusions:**

Our results align with the hypothesis of brain-first and body-first PD subtypes, providing novel insights into their different premotor non-motor burden and motor progression trajectories.

**Supplementary Information:**

The online version contains supplementary material available at 10.1007/s00415-025-13050-y.

## Introduction

Parkinson’s disease (PD) is a neurodegenerative condition characterized by both motor and non-motor symptoms. The clinical heterogeneity of PD has prompted efforts to define subtypes through motor phenotyping and data-driven models [1, 2]. Concurrently, postmortem staging systems have been developed to describe the distribution of intracellular α-synuclein, the pathological hallmark of PD, across different disease stages [3–5]. In an attempt to integrate these distinct staging and subtyping systems, the brain-first and body-first PD subtypes have recently been proposed [6]. In the brain-first subtype, α-synuclein accumulation and neurodegeneration begin centrally and progress cell-to-cell in a prion-like manner to the peripheral/enteric nervous system. Conversely, in the body-first subtype, peripheral α-synuclein pathology precedes central involvement.

REM sleep behavior disorder (RBD) is a non-motor symptom arising from neurodegeneration in the pons, which can precede or follow the onset of PD motor symptoms [7]. Premotor RBD has been proposed as a clinical hallmark of the body-first subtype as it precedes substantia nigra involvement in the midbrain (responsible for motor symptom onset) according to the caudo-rostral propagation model [8]. By contrast, RBD developing after the onset of motor signs should be consistent with the brain-first subtype.

If this hypothesis holds true, the body-first and brain-first subtypes would be expected to exhibit a different burden of non-motor symptoms before the onset of motor signs. However, information regarding the premotor non-motor burden in body-first versus brain-first subtypes is to date inconsistent. Additionally, it remains unclear whether the two subtypes differ in terms of motor severity and disease progression [9, 10].

In this study, we analyzed the natural history of the brain-first and body-first subtypes, providing a comprehensive perspective on both non-motor and motor domains. To this end, we retrospectively examined premotor non-motor symptoms at enrollment and conducted a prospective long-term motor assessment, using the attainment of Hoehn and Yahr (H&Y) stage 3 as a key milestone in PD progression [11, 12].

## Methods

### Study design

Patients attending the outpatient clinic of the Department of Neurology at the University Hospital of Bari (Italy) were consecutively enrolled over a 15-year period. All participants gave informed consent. PD diagnosis was made according to the UK PD Society Brain Bank criteria [13]. Patients were excluded if a comprehensive assessment revealed parkinsonism other than PD. Furthermore, patients with concomitant diseases associated with motor impairment, cognitive impairment, pain, or dysautonomia were also excluded. All assessments were performed by a specialist in movement disorders with expertise in parkinsonism.

### Baseline assessment

At enrollment, a semi-structured questionnaire was used to collect information on age, sex, education, family history of parkinsonism, current antiparkinsonian treatment, age at onset of motor symptoms, motor phenotype at onset (including the presence of tremor and bilateral motor symptoms), and non-motor symptoms.

Levodopa equivalent daily dose (LEDD) was determined using established methodologies [14].

RBD was diagnosed using the RBD1Q, which provides a probable RBD diagnosis [15], and the age of RBD onset was recorded. Hyposmia was evaluated using a single screening question about smelling ability problems [16]. Subjective cognitive symptoms were explored using the Unified Parkinson’s Disease Rating Scale (UPDRS) part I, question 1 [17]. Since this question primarily addresses memory loss, disorientation, and executive deficits, additional cognitive issues—such as difficulty maintaining concentration, language problems, and visuospatial deficits—were also recorded if suggested by patients or caregivers. Psychiatric symptoms (hallucinations, depression, anxiety, and apathy) were assessed using the UPDRS part I, questions 2–4 [17]. Hallucinations occurring only during sleep–wake transitions were excluded. Other psychiatric symptoms, such as delirium, emotional lability, nervousness, impulsivity/compulsivity, and aggressive behaviors, were also recorded. Pain was assessed using the UPDRS part II, question 17 [17]. Constipation was defined according to the ROME III diagnostic criteria [18]. Other dysautonomic symptoms (orthostatic hypotension, urinary, sexual, and sweating dysfunctions) were explored using the Unified Multiple System Atrophy Rating Scale part I, questions 9–11 [19], and the UPDRS part II, question 6 [17]. In patients reporting orthostatic symptoms, orthostatic hypotension was confirmed by manually measuring blood pressure after 5 min in the supine position and at 1 and 3 min after standing. Orthostatic hypotension was defined as a sustained decrease in systolic blood pressure of at least 20 mmHg or in diastolic blood pressure of at least 10 mmHg within 3 min of standing, compared to the blood pressure measured in the supine position [20].

All non-motor symptoms and domains were documented as “present” or “absent”, including information on whether they occurred before or after the onset of parkinsonian motor symptoms.

### Follow-up

Following the baseline assessment, patients were prospectively monitored, with follow-up visits conducted twice a year. Long-term motor prognosis was assessed by determining the achievement of H&Y stage 3 during the on-medication state [11].

### Statistical analysis

Statistical analysis was carried out using the SPSS software package (version 23.0). Data were expressed as mean ± standard deviation (SD) unless otherwise indicated. Normality of data was assessed with Shapiro–Wilk and Kolmogorov–Smirnov tests and Q–Q plots. According to the brain-first and body-first hypothesis, patients were divided into two groups: patients with probable premotor RBD (PD^preRBD+^) and patients without probable premotor RBD (PD^preRBD−^). Differences between groups were examined using the chi-square test for categorical variables and the Mann–Whitney U test for continuous variables. Odds ratios (ORs) and corresponding 95% confidence intervals (CIs) were estimated using logistic regression analysis and adjusted for relevant variables. Finally, the time to reach H&Y stage 3 was estimated using Kaplan–Meier curves and Cox regression analysis. Study time was represented by the time elapsed between motor onset and the achievement of H&Y stage 3. Patients who did not reach H&Y stage 3 during the observation period were included in the survival analysis for the entire duration of their follow-up. Patients who had already reached H&Y stage ≥ 3 at enrollment were excluded from survival analysis. Kaplan–Meier curves were compared using the log-rank test, while hazard ratios (HRs) and corresponding 95% CIs were computed for the purposes of Cox regression analysis. Significance was set at the 0.05 level.

## Results

### Study population

We enrolled 400 PD patients (242 men and 158 women) aged 66.0 ± 9.6 years. PD duration since the onset of motor symptoms was 4.0 ± 4.1 years. Their demographic and clinical characteristics are summarized in Table 1.

**Table 1:** Demographic and motor phenotype of the study population at enrollment

	Overall population	PD^preRBD+^ (*N* = 81)	PD^preRBD−^ (*N* = 319)	*P* value^a^	Odds ratio (95% CI)^b^
Age at enrollment, mean years (SD)	66.0 (9.6)	67.9 (8.0)	65.5 (9.9)	0.07	1.03 (1.00 to 1.06)
Men, *n* (%)	242 (60.5)	55 (67.9)	187 (58.6)	0.1	1.49 (0.89 to 2.50)
Years of schooling, mean (SD)	8.8 (4.6)	8.8 (5.1)	8.8 (4.4)	0.7	1.00 (0.94 to 1.06)
Family history of parkinsonism, *n* (%)	52 (13.0)	12 (14.8)	40 (12.5)	0.6	1.21 (0.60 to 2.44)
LEDD at enrollment, mean mg (SD)	346.3 (327.6)	337.7 (292.8)	348.5 (336.6)	> 0.99	1.00 (0.999 to 1.001)
Age at motor onset, mean years (SD)	62.0 (9.6)	64.9 (8.1)	61.2 (9.8)	0.002	1.04 (1.02 to 1.07)
Tremor at motor onset, *n* (%)	262 (65.5)	43 (53.1)	219 (68.7)	0.008	0.52 (0.31 to 0.85)
Bilateral motor symptoms at motor onset, *n* (%)	66 (16.5)	23 (28.4)	43 (13.5)	0.001	2.55 (1.42 to 4.55)

^a^The *P* values were estimated using the chi-square test for categorical variables and the Mann–Whitney *U* test for continuous variables

^b^Odds ratios with corresponding 95% CIs were estimated using univariable logistic regression

*PD* Parkinson’s disease, *PD*^*RBD+*^ PD patients with probable premotor RBD, *PD*^*RBD−*^ PD patients without probable premotor RBD, *CI* confidence interval, *SD* standard deviation, *LEDD* levodopa equivalent daily dose

At enrollment, 149 (37.3%) patients were diagnosed with probable RBD, 121 (30.3%) reported hyposmia, 86 (21.5%) subjective cognitive symptoms, 146 (36.5%) psychiatric symptoms, 108 (27.0%) pain, 138 (34.5%) constipation, and 60 (15.0%) other dysautonomic symptoms.

Among the 149 patients who were diagnosed with RBD, 81 reported premotor RBD (mean age at RBD onset, 59.9 ± 10.9 years) and 68 had post-motor RBD (mean age at RBD onset, 66.0 ± 7.0 years).

Patients with premotor RBD (PD^preRBD+^) were compared with patients with post-motor RBD and without RBD combined (PD^preRBD−^). PD^preRBD+^ and PD^preRBD−^ patients (*n* = 81 vs. 319) did not significantly differ in terms of age, sex, education, family history of parkinsonism, and LEDD. At motor onset, however, PD^preRBD+^ patients were significantly older and reported a lower frequency of tremor and a higher frequency of bilateral motor symptom onset than PD^preRBD−^ patients (Table 1).

Among the 319 PD^preRBD−^ patients, those who reported post-motor RBD were comparable to those without RBD for demographic and clinical features (Supplemental Table 1).

### Premotor symptoms

Compared with PD^preRBD−^, PD^preRBD+^ patients reported a higher frequency of hyposmia, cognitive symptoms, pain, constipation, and other dysautonomic symptoms before the appearance of parkinsonian motor symptoms. Among premotor dysautonomic manifestations, urinary symptoms were reported by 8 PD^preRBD+^ and 11 PD^preRBD−^ patients, orthostatic hypotension was documented in 1 PD^preRBD+^ and 3 PD^preRBD−^ patients, and sexual dysfunction was reported by 4 PD^preRBD+^ patients. No significant difference was observed between the two groups in terms of premotor psychiatric symptoms (Table 2).

**Table 2:** Burden of premotor non-motor symptoms in PD patients with and without premotor REM sleep behavior disorder (RBD)

	Overall population	PD^preRBD+^ (*N* = 81)	PD^preRBD−^ (*N* = 319)	Adjusted odds ratio (95% CI),* P* value^a^
Hyposmia, *n* (%)	97 (24.3)	38 (46.9)	59 (18.5)	3.86 (2.12 to 7.05), 0.00001
Cognitive symptoms, *n* (%)	20 (5.0)	10 (12.3)	10 (3.1)	3.22 (1.06 to 9.72), 0.04
Psychiatric symptoms, *n* (%)	77 (19.3)	22 (27.2)	55 (17.2)	1.61 (0.83 to 3.10), 0.2
Pain, *n* (%)	64 (16.0)	22 (27.2)	42 (13.2)	2.02 (1.02 to 4.03), 0.045
Constipation, *n* (%)	93 (23.3)	34 (42.0)	59 (18.5)	2.28 (1.23 to 4.22), 0.009
Other dysautonomic symptoms, *n* (%)	26 (6.5)	12 (14.8)	14 (4.4)	3.77 (1.59 to 8.94), 0.003

^a^Adjusted for sex, years of schooling, age at motor onset, and motor disease duration at recruitment using logistic regression analysis

*PD* Parkinson’s disease, *PD*^*RBD+*^ PD patients with probable premotor RBD, *PD*^*RBD−*^ PD patients without probable premotor RBD, *CI* confidence interval, *SD* standard deviation

Among the 319 PD^preRBD−^ patients, those who reported post-motor RBD were comparable to those without RBD for distribution of premotor non-motor symptoms (Supplemental Table 1).

### Motor progression

**Fig. 1:**
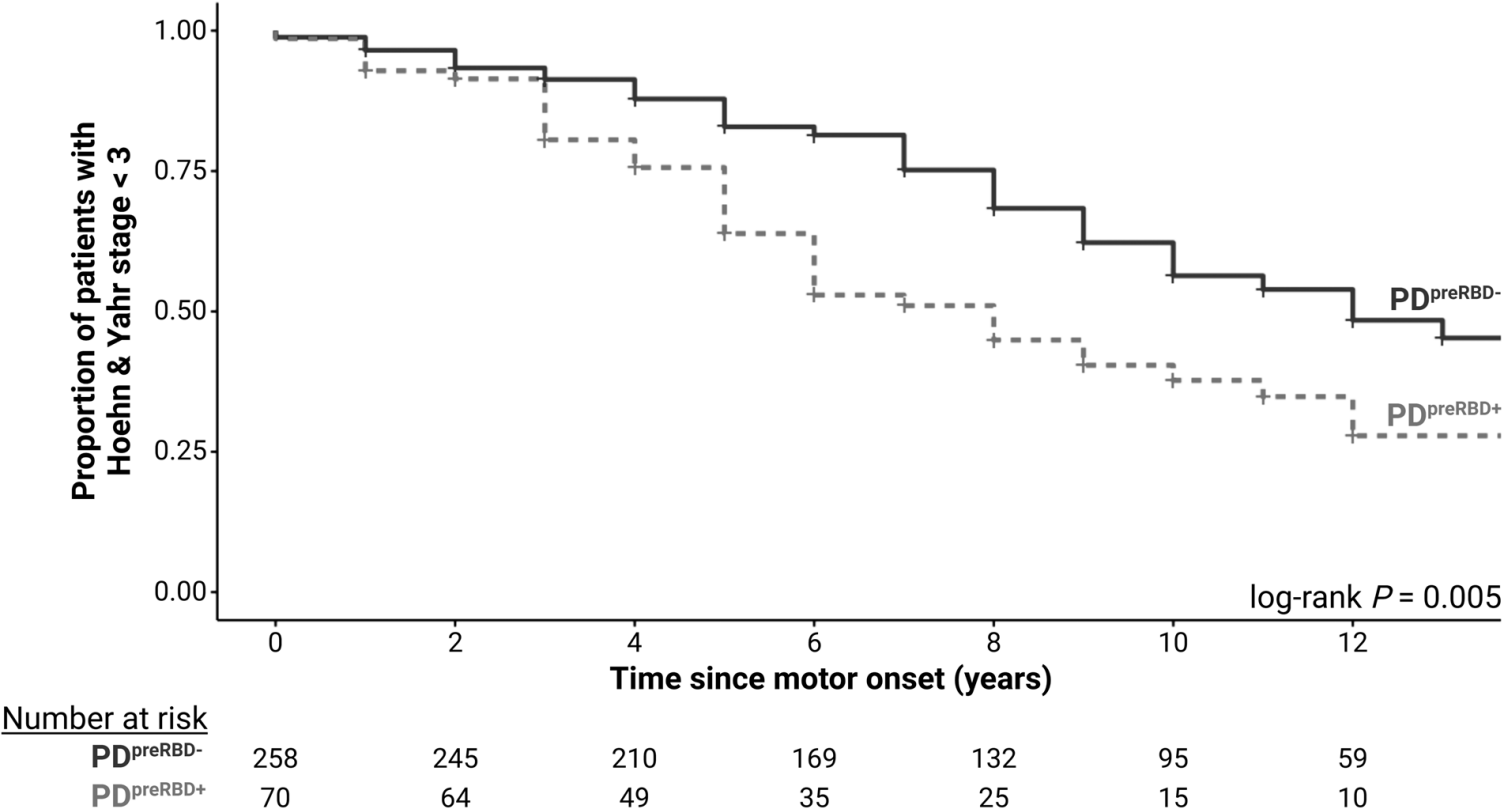
Kaplan–Meier plots of survival to Hoehn and Yahr stage 3 in patients with and without premotor RBD (PD^preRBD+^ and PD^preRBD−^, respectively)

At enrollment, 72 patients (11/81 with PD^preRBD+^ vs. 61/319 with PD^preRBD−^, *P* = 0.2) had already reached H&Y stage ≥ 3 and were therefore excluded from the subsequent analysis. In the remaining patients (70 with PD^preRBD+^ and 258 with PD^preRBD−^), motor progression was prospectively evaluated for up to 9.7 ± 4.9 years (8.6 ± 4.3 years in PD^preRBD+^ patients vs. 10.0 ± 5.1 years in PD^preRBD−^ patients, *P* = 0.05). PD^preRBD+^ patients exhibited a more rapid progression to H&Y stage 3 than PD^preRBD−^ patients (Fig. 1, log-rank *P* = 0.005). On Cox regression analysis (Table 3), the significant association of PD^preRBD+^ and H&Y stage 3 persisted even after adjusting for sex, years of schooling, age at motor onset, and initial motor phenotype (presence of tremor and bilateral motor symptoms). The mean age at H&Y stage 3 was similar in the two groups (70.9 ± 7.7 years in PD^preRBD+^ patients vs. 70.5 ± 8.5 years in PD^preRBD−^ patients, *P* = 0.9).

**Table 3:** Univariable and multivariable Cox regression analysis of predictor variables for progression to Hoehn and Yahr stage 3

	Univariable model hazard ratio (95% CI), *P* value	Multivariable model hazard ratio (95% CI), *P* value
Premotor RBD	1.64 (1.14 to 2.37), 0.007	1.63 (1.07 to 2.47), 0.02
Sex (F vs. M)	1.41 (1.03 to 1.93), 0.04	1.43 (0.99 to 2.07), 0.06
Years of schooling	0.96 (0.92 to 1.004), 0.08	1.00 (0.96 to 1.05), 0.9
Age at motor onset	1.07 (1.05 to 1.10), < 0.00001	1.07 (1.04 to 1.10), < 0.00001
Tremor at onset	0.81 (0.58 to 1.13), 0.2	0.83 (0.57 to 1.22), 0.2
Bilateral motor symptoms at onset	1.91 (1.26 to 2.91), 0.002	1.18 (0.72 to 1.94), 0.5

*CI* confidence interval

Among the 319 PD^preRBD−^ patients, those who reported post-motor RBD were comparable to those without RBD for motor progression to H&Y stage 3 (data not shown).

## Discussion

Compared with PD^preRBD−^ patients, PD^preRBD+^ patients developed parkinsonian motor symptoms later, exhibited a greater burden of premotor non-motor symptoms (specifically hyposmia, cognitive symptoms, pain, constipation, and dysautonomia), and showed faster progression to H&Y stage 3.

The older age at motor symptom onset in the PD^preRBD+^ group is consistent with a previous report [8], and may reflect pathophysiological differences between the two PD subtypes, differences in the timing of biological disease onset, or differences in the duration of the premotor phase. Indeed, PD^preRBD+^ patients might experience a longer premotor phase due to the prolonged time required for the propagation of synucleinopathy from the peripheral/enteric nervous system to the midbrain.

Although several previous studies have documented a higher non-motor burden in PD patients with RBD [21–29], these studies either did not stratify patients based on the presence of premotor RBD or did not specifically examine premotor non-motor symptoms. The higher burden of specific non-motor symptoms (including hyposmia, cognitive impairment, pain, constipation, and dysautonomia) we observed in the premotor phase of patients with premotor RBD provides novel evidence supporting the hypothesis of PD subtypes with distinct pathological origins, namely body-first and brain-first subtypes.

PD^preRBD+^ and PD^preRBD−^ patients also exhibited differences in their motor features. The lower prevalence of tremor and the greater occurrence of bilateral motor symptoms at onset we detected in PD^preRBD+^ patients align with previous studies [9, 23, 30, 31], and could be consistent with a worse prognosis. Indeed, tremor-dominant onset is regarded as a predictor of a more benign disease course, whereas symmetrical motor symptom onset has been more frequently associated with a “malignant” phenotype [1, 31–35]. The association between premotor RBD and a faster progression to H&Y stage 3 was independent of tremor, bilateral motor symptoms at onset, and other potentially confounding factors. This is a novel finding as the existing literature has provided conflicting evidence on whether the body-first and brain-first subtypes differ in terms of motor progression [9, 10, 27, 36]. While previous studies were limited by several biases, the robustness of our findings is strengthened by information on the temporal relationship between RBD and motor symptom onset, longitudinal follow-up, and an adequately sized PD sample.

This study has strengths and limitations. The absence of polysomnographic confirmation for RBD could reduce the diagnostic accuracy, increasing the risk of false negatives and, more notably, false positives, as other sleep disorders can mimic RBD [37]. However, the RBD1Q has been demonstrated to have relatively high sensitivity (93.8%, with 95% CI 90.0 to 96.2) and specificity (87.2%, with 95% CI 82.4 to 90.8) in detecting RBD [15], and the frequency of RBD in our sample (37.3%) was in line with the literature, which reports an average incidence of 42.3% in PD [38]. Nonetheless, the possibility of misclassification in categorizing patients as PD^preRBD+^ or PD^preRBD−^ cannot be entirely ruled out. To assess non-motor symptoms, we employed concise tools instead of more extensive rating scales. These tools had the advantage of being applicable retrospectively while maintaining good accuracy. Coherently, the frequency of non-motor symptoms was consistent with those reported in other larger PD cohorts (see Supplemental Table 2) [39, 40]. Due to this approach, however, some non-motor symptoms, as well as the overall severity of non-motor symptoms, were not investigated. We did not include an objective olfaction test such as the University of Pennsylvania Smell Identification Test (UPSIT) [41] or the “Sniffin’ Sticks” [42]. This may have led to an underestimation of hyposmia frequency, even considering that many PD patients may be unaware of their hyposmia [43]. However, the finding that more PD^preRBD+^ patients were aware of hyposmia suggests a higher frequency and/or severity of hyposmia in the body-first subtype, as previously demonstrated [8, 9]. The single-center design may introduce some biases. However, it also ensured standardized assessments for all patients over time and the reproducibility of evaluations. The consecutive recruitment of patients reduced the risk of selection bias, and the large sample size provided satisfactory statistical power. Retrospective data collection at enrollment might have been biased in terms of the accuracy of some information regarding the premotor phase. However, our protocol required informed family members and, where available, past medical records to confirm the patient’s information. In addition, the retrospective assessment was conducted after an average of only 4 years from motor onset, which reduced the risk of recall bias in classifying non-motor symptoms as premotor or post-motor and in defining motor onset phenotype. In contrast, assessment of motor progression was conducted prospectively, ensuring a high level of accuracy. Lastly, we used H&Y staging rather than the UPDRS part III as the outcome measure for motor progression as H&Y staging is less susceptible to confounding by antiparkinsonian treatment [44].

**Fig. 2:**
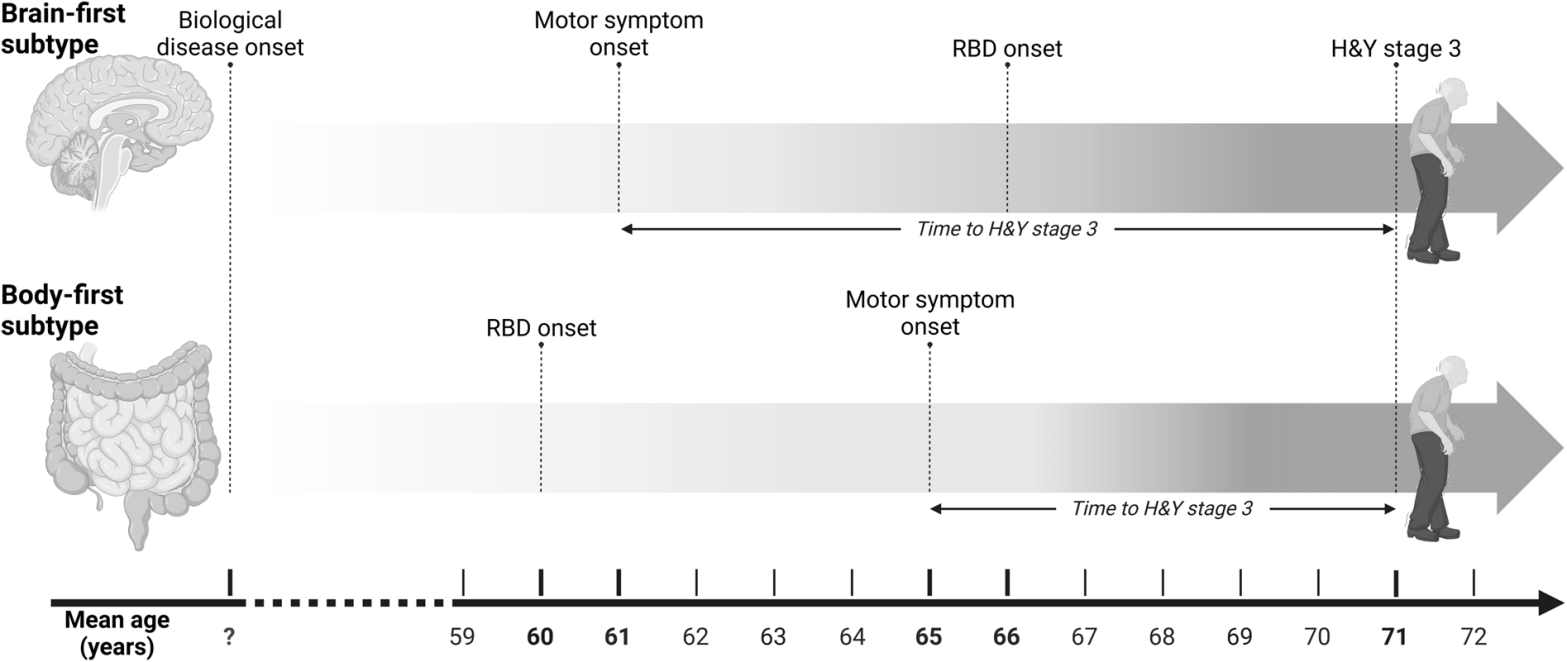
The natural history of body-first versus brain-first Parkinson’s disease subtypes

Despite the foregoing limitations, our findings provide new evidence supporting the hypothesis of brain-first and body-first PD subtypes, along with novel information on their natural history (Fig. 2). The observed differences in premotor non-motor burden between distinct sites of PD pathology onset may reflect pathophysiological differences and/or differences in the time elapsing between biological disease onset and the appearance of motor signs. The faster progression to H&Y stage 3 in PD^preRBD+^ patients may suggest a more “aggressive” phenotype of PD or a more advanced biological stage at disease motor onset in this subgroup. Regardless of the interpretation, premotor RBD emerged as an independent predictive factor for motor progression. Its influence on the burden of premotor non-motor symptoms and on motor progression was highly specific as it was not observed in patients who developed post-motor RBD. Identifying brain-first and body-first PD subtypes at diagnosis may have important implications for prognosis communication and initial treatment selection.

## Supplementary Information

Below is the link to the electronic supplementary material.

Supplementary file1 (DOCX 35 KB)

## Data Availability

Data are available upon reasonable request. Requests for data sharing can be sent to the corresponding author, Vittorio Velucci, at the e-mail address vittoriovelucci@gmail.com.
