# Supplementary material for: The natural history of body-first versus brain-first Parkinson’s disease subtypes
Source: J Neurol. 2025 Apr 9;272(5):325. doi: 10.1007/s00415-025-13050-y (PMC11982158; doi:10.1007/s00415-025-13050-y)
Supplement: Supplementary file 1 — Supplementary file1 (DOCX 35 KB) [file 415_2025_13050_MOESM1_ESM.docx]

| **Supplemental Table 1.**  Demographic and clinical characteristics of patients with post-motor RBD and those without RBD at study enrolment. | | | |
| --- | --- | --- | --- |
|  | **PD with post-motor RBD**  **(N = 68)** | **PD without RBD at enrolment**  **(N = 251)** | ***P*-value^a^** |
| **Demographic characteristics** |  |  |  |
| Men, n (%) | 45 (66.2) | 142 (56.6) | 0.2 |
| Years of schooling, mean (SD) | 8.7 (4.8) | 8.8 (4.3) | 0.8 |
| Family history of parkinsonism, n (%) | 11 (16.2) | 29 (11.6) | 0.3 |
| Age at motor onset, mean years (SD) | 62.3 (8.2) | 60.9 (10.2) | 0.5 |
| **Motor phenotype at onset** |  |  |  |
| Tremor at onset, n (%) | 48 (70.6) | 171 (68.1) | 0.7 |
| Bilateral motor symptoms at onset, n (%) | 8 (11.8) | 35 (13.9) | 0.6 |
| **Premotor symptoms** |  |  |  |
| Hyposmia, n (%) | 15 (22.1) | 44 (17.5) | 0.4 |
| Cognitive symptoms, n (%) | 3 (4.4) | 7 (2.8) | 0.5 |
| Psychiatric symptoms, n (%) | 8 (11.8) | 47 (18.7) | 0.2 |
| Pain, n (%) | 4 (5.9) | 38 (15.1) | 0.05 |
| Constipation, n (%) | 13 (19.1) | 46 (18.3) | 0.9 |
| Other dysautonomic symptoms, n (%) | 1 (1.5) | 13 (5.2) | 0.2 |
| ^a^The *P*-values were estimated using the chi-square test for categorial variables and the Mann-Whitney U test for continuous variables. | | | |

| **Supplemental Table 2.** Frequency of non-motor symptoms at enrolment in our study population compared with two other Parkinson’s disease cohorts. | | | |
| --- | --- | --- | --- |
|  | **Study population** | **PRIAMO cohort**  **(Barone et al. 2009)** | **SYNAPSES cohort**  **(Fernandes et al. 2021)** |
| **Sample characteristics** |  |  |  |
| Number of patients, n | 400 | 1072 | 1589 |
| Men, n (%) | 242 (60.5) | 647 (60.4) | 978 (61.6) |
| Age, mean years (SD) | 66.0 (9.6) | 67.4 (9.4) | 68.4 (9.6) |
| Disease onset age, mean years (SD) | 62.0 (9.6) | 61.0 (10.6) | NA |
| Disease duration, median years (IQR) | 3.0 (1.0-5.0) | 5.1 (2.8-9.1) | NA |
| Years from diagnosis, mean years (SD) | 2.9 (3.9) | NA | 7.9 (5.4) |
| **Frequency of non-motor symptoms** |  |  |  |
| Hyposmia | 30.3% | 26.9% | NA |
| Cognitive | 21.5%^a^ | 44.7%^b^ | 16.7% |
| Psychiatric | 36.5% | 66.8% | 43.0% |
| Pain | 27.0% | 60.9% | 20.9% |
| Constipation | 34.5% | 27.5% | NA |
| Urinary | 12.8% | 57.3% | 22.1% |
| Cardiovascular | 2.3%^c^ | 14.7% | 9.4% |
| ^a^Subjective cognitive symptoms.  ^b^Cognitive impairment was defined as a MMSE age- and education-adjusted score ≤ 23.8 and/or FAB age- and education-adjusted score ≤ 13.48, according to Italian normative data.  ^c^Objectively confirmed symptomatic orthostatic hypotension.  Abbreviations: NA = not available. | | | |
